# Reassortment incompetent live attenuated and replicon influenza vaccines provide improved protection against influenza in piglets

**DOI:** 10.1038/s41541-024-00916-x

**Published:** 2024-07-13

**Authors:** Annika Graaf-Rau, Kathrin Schmies, Angele Breithaupt, Kevin Ciminski, Gert Zimmer, Artur Summerfield, Julia Sehl-Ewert, Kathrin Lillie-Jaschniski, Carina Helmer, Wiebke Bielenberg, Elisabeth grosse Beilage, Martin Schwemmle, Martin Beer, Timm Harder

**Affiliations:** 1 Institute of Diagnostic Virology, Friedrich-Loeffler-Institut, Greifswald-Insel Riems, Greifswald, Germany; 2 Field Station for Epidemiology, University of Veterinary Medicine Hannover, Foundation, Bakum, Germany; 3 Department of Experimental Animal Facilities and Biorisk Management, Friedrich-Loeffler- Institut, Greifswald, Insel Riems, Germany; 4 Institute of Virology, Medical Center University of Freiburg, Freiburg, Germany; 5 Faculty of Medicine, University of Freiburg, Freiburg, Germany; 6 Institute of Virology and Immunology, Bern & Mittelhaeusern, Switzerland, and Department of Infectious Diseases and Pathobiology, Vetsuisse Faculty, University of Bern, Bern, Switzerland; 7 Ceva Santé Animale, Duesseldorf, Germany; 8 SAN Group Biotech Germany GmbH, Hoeltinghausen, Germany

**Keywords:** Infectious diseases, Respiratory tract diseases

## Abstract

Swine influenza A viruses (swIAV) cause an economically important respiratory disease in modern pig production. Continuous virus transmission and antigenic drift are difficult to control in enzootically infected pig herds. Here, antibody-positive piglets from a herd enzootically infected with swIAV H1N2 (clade 1 A.3.3.2) were immunized using a homologous prime-boost vaccination strategy with novel live attenuated influenza virus (LAIV) based on a reassortment-incompetent bat influenza-swIAV chimera or a vesicular stomatitis virus-based replicon vaccine. Challenge infection of vaccinated piglets by exposure to H1N2 swIAV-infected unvaccinated seeder pigs showed that both LAIV and replicon vaccine markedly reduced virus replication in the upper and lower respiratory tract, respectively, compared to piglets immunized with commercial heterologous or autologous adjuvanted whole-inactivated virus vaccines. Our novel vaccines may aid in interrupting continuous IAV transmission chains in large enzootically infected pig herds, improve the health status of the animals, and reduce the risk of zoonotic swIAV transmission.

## Introduction

Swine influenza viruses (swIAVs) affect feral and domestic pigs across the world and can infect pigs at any age, including neonates^1^. The hemagglutinin (HA) subtypes H1 or H3 and the neuraminidase (NA) subtypes N1 or N2 are prevailing in currently circulating swIAVs. For each of the HA subtypes, multiple viral lineages exist which are classified as avian (av or 1C), human (hu or 1B), or pandemic (pdm or 1A), depending on the initial host they were isolated from and their association with phylogenetic clades^2^. Human seasonal influenza A viruses (IAVs) are closely related to swIAVs. In fact, reciprocal transmissions across the porcine-human interface play a major role in expanding swIAV diversity in pig populations^3^ and, in turn, bear a pandemic threat to the human population^4^. Pigs are also susceptible to infection by IAV of avian origin and may serve as “mixing vessels” or intermediate influenza virus hosts, which may allow the reassortment of genome segments of avian, porcine, and human IAVs creating widespread prospects for zoonotic transmissions of reassorted viruses with unpredictable pandemic potential^5^. Pig multiplying and fattening infrastructures have been expanding considerably in size over the last 50 years. Sizable swine herds exceeding 3000 individuals, which feature high population turnover rates, are capable of maintaining enzootic, self-sustaining swIAV infections following virus incursions^6^. Indeed, for several years now, an enzootic form of influenza has been described in large pig herds, which insidiously and continuously compromises pig health^7^. These holdings also harbor a vast and complex reservoir of swIAV with herd-specific antigenicity, zoonotic, and even pre-pandemic potential in European, Asian, and American pig populations^8–12^.

Currently, there are no legal requirements in Europe for the reporting of swIAV infections in pigs, and official, coordinated monitoring plans and control measures are mostly lacking. Apart from biosecurity measures and herd management, vaccination of sows using either commercial or autologous adjuvanted whole-inactivated vaccines (WIV) is frequently used to control swIAV infections. In addition, suckling piglets might receive a certain level of immune protection through the transfer of maternally derived antibodies (MDA)^13^. Yet, in pig holdings that are persistently affected by swIAV, MDA-positive piglets at a young age, and even neonates during their first week of life, have been found to function as asymptomatic amplifiers of swIAV although productive infection does not induce active humoral immunity measurable as an increase of specific antibodies in the presence of MDA^1,14–17^. Likewise, WIV-based induction of active swIAV immunity in piglets has been shown to be hampered by interference with MDA, and, as a result, commercially licensed WIV vaccines, in Europe, are only approved for use in pigs above 56 days of age^1,18^. MDA interference with vaccinations in neonates and very young mammals including pigs have tentatively been traced to increased immunosuppressive activity of Treg cells triggered by maternally derived IgG^19^ and to hampering B cell differentiation in germinal centers^20^. Commercial and autologous WIV are effective in reducing viral lung loads, shedding, and the severity of clinical signs^21^. Their efficacy, however, is increasingly compromised by expanding virus diversity and antigenic drift which is increasing in continuously infected large herds^22–24^. WIVs, in general, induce a highly strain-specific humoral immune response; adjuvants and frequent re-vaccinations may foster the breadth of antibody and T cell responses^25^. Nevertheless, updates of vaccine antigens may be required in order to cover the antigenic gaps to currently circulating field viruses^21,26–28^.

New swIAV vaccine candidates and vaccination concepts have been developed with the aim to induce a generally broader immunity including a strong T cell response and mucosal defense mechanisms. Many attempts focused on live-attenuated influenza vaccines (LAIV)^29–31^. These vaccines replicate in the host but without causing disease while mimicking natural infection, stimulating a comprehensive, robust, long-lasting and, ideally, cross-protective immunity that relies on humoral and cellular immune effectors in a systemic and/or mucosal fashion^31^. Recently, we have shown that chimeric bat-IAV that express both the HA and NA of the desired target IAV on the backbone of either H17- or H18-derived bat-IAV represent a promising approach as they replicate in a variety of avian and mammalian hosts following intranasal inoculation. Due to incompatibilities of the bat-IAV genome segment ends, reassortment with other mammalian and avian IAV is blocked, thus, preventing reversion of virulence due to reassortment^32–35^.

An alternative strategy employs RNA replicon particle vaccines based on propagation-defective vesicular stomatitis virus (VSV) vectors^36^. Due to the affinity of the VSV glycoprotein (G) for the low-density lipoprotein receptor, it is able to infect and replicate in a variety of tissues, thereby eliciting strong humoral and cellular immune responses^37^. Recent other studies have shown that a propagation-incompetent VSV vector encoding HA H7 or H5 protects chickens from challenge infection with HPAIV of subtype H7 or H5^38–40^.

The present study investigates the efficacy of replicon and chimeric bat-IAV vaccines matched to a homologous swIAV isolate in a prime-boost vaccination-challenge setting in young piglets from an enzootically swIAV-infected pig farm. Results are compared to age-matched syntopic piglet groups vaccinated with a commercially available heterologous or an autologous WIV.

## Results

### Case setting

A conventional indoor piglet production farm in Western Germany of approximately 700 sows had previously been diagnosed with enzootic swIAV infections of the H1pdmN2 lineage; respiratory disease associated with continuous detection of subtype H1pdmN2 infections has been smoldering for more than one year. Piglets are produced in a 3-week batch farrowing rhythm and all sows are quarterly vaccinated against swIAV with the commercial WIV vaccines Respiporc® FLU3 and Respiporc® FLUpan H1N1 (Ceva Santé Animale, Libourne, France). In the frame of a longitudinal study in this persistently infected swine holding, a number of swIAV-positive swabs have been obtained and used for full-genome sequencing and virus isolation. The H1pdmN2 virus isolates AI03362, belonging to clade 1A.3.3.2/pdm (II-like), was dominating in this holding for several months. This strain was obtained, passaged twice in swine testicle (ST) cells, fully sequenced (accession number EPI_ISL_17646249), and titrated on MDCK II cells. This isolate was used for the generation of VSV replicon and bat-flu chimera LAIVs, autologous WIV, and also as a challenge virus.

### Selection, design, and application of vaccines

In the affected holding, routine sow vaccination alone was not sufficient to interrupt continuous swIAV circulation. In order to enhance the immunity of the pigs and to silence a potentially important swIAV amplification reservoir, we pursued to induce protection in piglets from an early age on. Therefore, we designed two vaccines based on the HA and NA sequences of the H1pdmN2 AI03362 virus isolate. (i) *VSV replicon vaccine*: The HA and NA open reading frames were separately inserted into the vesicular stomatitis virus (VSV) genome thereby replacing the VSV glycoprotein (G) gene. The resulting vector vaccine candidates VSVΔG(H1) and VSVΔG(N2) were propagated on helper cells providing the VSV G protein in trans. (ii) The LAIV *bat-IAV* carried the same HA and NA sequences in a single chimeric virus; it was successfully rescued, plaque-purified, and propagated on MDCK cells.

In addition, two WIV approaches were selected. First, a combination of two commercial products registered by the European Medicines Agency (EMA), a trivalent heterologous vaccine (Respiporc FLU3, Ceva Santé Animale, Libourne, France), containing antigens of H1avN1 (clade 1C), H1huN2 (clade 1B) and H3N2, and a monovalent vaccine (Respiporc FLUpan H1N1, Ceva Santé Animale, Libourne, France) containing H1pdmN1 (clade 1A) were used simultaneously in an off-label fashion, i.e., before day 56 of life. Furthermore, an autologous WIV was produced according to good manufacturing practise (GMP) conditions featuring the abovementioned homologous H1pdmN2 isolate. The autologous vaccine was designed for a dosage of 1 ml/animal containing >256 hemagglutination units/ml (HAU/ml) before inactivation. A polymer was used as an adjuvant (Ictyolane 50®, IctyoDev, France). Under the same conditions, a polymer-adjuvanted mock vaccine was produced using the cell-culture supernatant of uninfected ST cells.

**Fig. 1:**
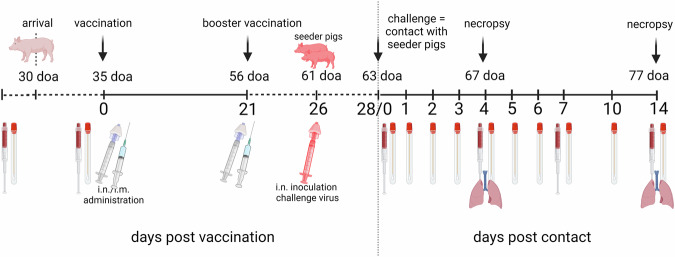
Experimental study design. Pigs were vaccinated twice at 35 and 56 days of age (doa), respectively, and challenged at 63 doa. Challenge was performed by direct contact with two non-vaccinated, age-matched syntopic seeder pigs (shown in red) per group which had been inoculated intranasally with swIAV isolate H1pdmN2 (AI03362) using a dose of 10^6^ TCID_50_ per animal at 61 doa. One seeder animal and four vaccinees per group were sacrificed for necropsy at day 4 post contact challenge (dpc). The remaining animals were kept until 14 dpc. Blood samples and nasal swabs were taken as indicated by the symbols. This figure was created with BioRender.com and licensed by the company under agreement number CL26YA977R.

A prime-boost vaccination scheme was applied starting at 35 days of age for all vaccines (Fig. 1). The booster vaccination was given at 56 days of age and the challenge infection at 63 days of age. Except for the bat-IAV chimeric vaccine, which was applied intranasally using a mucosal atomization device (Wolfe Tory Medical, USA) all other vaccines were injected intramuscularly into the neck at the highest point of the ear base. None of the vaccines induced adverse reactions at the local injection site or after nasal application (bat-IAV chimera) nor any clinical signs in the follow-up. Body temperature remained normal in all piglets prior to challenge infection (not shown).

### Direct contact with infected seeder pigs ensured efficient viral transmission to vaccinees and controls

**Fig. 2:**
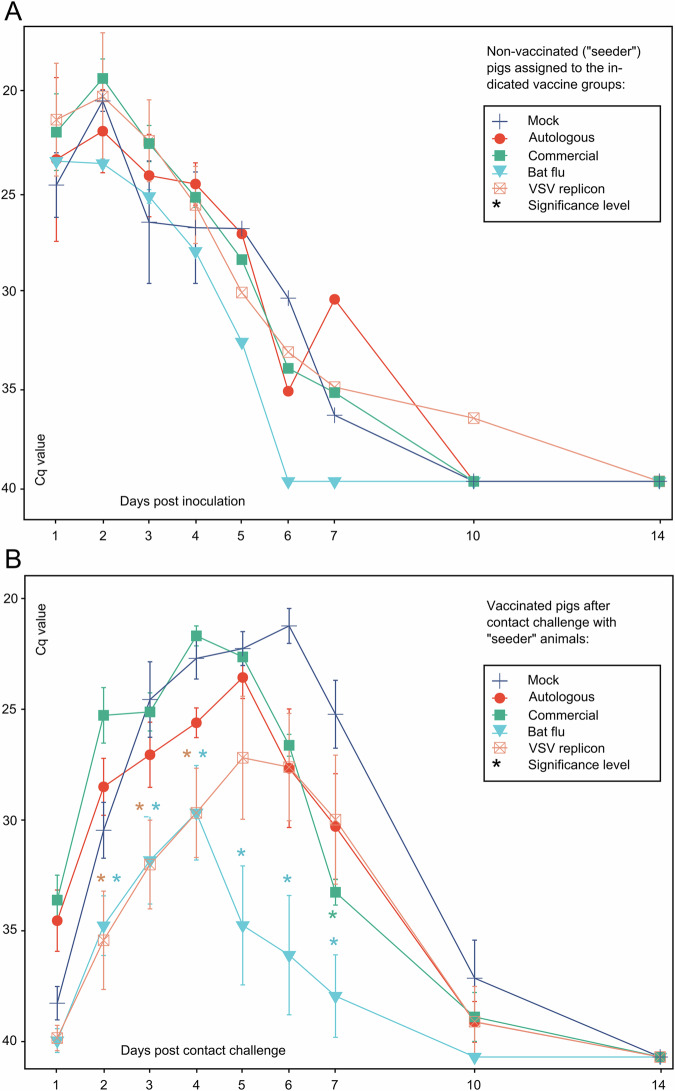
Virus excretion in nasal swabs. Excretion of viral RNA is shown for **A** non-vaccinated seeder piglets that had been individually inoculated with swIAV isolate H1pdmN2 (AI03362) by the nasal route and **B** vaccinated piglets after contact exposure to seeders. Two seeder pigs each were assigned to eight vaccinees per group at 2 dpi; the color of the seeder pig pairs in **A** indicates to which vaccine group they were associated. Viral loads were measured by an M-specific RT-qPCR; Cq values are indicated on the Y-axis. Error bars indicate the standard deviation of the median. Asterisks indicate highly significant differences (*p* < 0.01) of indicated group from Mock group (Supplementary Table 2; Kruskal–Wallis with Dunn–Bonferoni correction).

To mimic realistic virus transmission scenarios in pig holdings, 7 days after the booster immunization vaccinees were exposed to non-vaccinated pigs which had been individually inoculated intranasally with 10^6^ TCID_50_ of swIAV isolate AI03362 of subtype H1pdmN2. These so-called “seeder pigs” developed a highly productive swIAV infection as shown by virus excretion patterns in nasal swabs (Fig. 2A). Viral RNA excretion in the seeder pigs subsided at about day 7 post-inoculation (dpi). At 2 dpi when virus excretion peaked, two seeders were assigned to each of the five vaccine groups. Judging from the fairly homogenous viral RNA loads nasally excreted by the seeders during 1–4 dpi, it can be assumed that virus exposure of the vaccinees by contact with seeders was reasonably similar across the groups. In the immunized contact animals, productive swIAV infection developed from day 2 post contact challenge (dpc) as seen by nasal excretion dynamics of viral RNA (Fig. 2B). Some animals even revealed viral RNA on 1 dpc. No clinical signs of disease attributable to H1pdmN2 infection were recorded in seeder pigs or vaccinated in-contact piglets including the mock-vaccinated group. Also, rectal temperatures remained fairly stable between 39-40°C throughout the observation period (Supplementary Fig. 1).

### LAIV and VSV replicon vaccine efficiently reduced nasal excretion of viral RNA

Based on nasal virus excretion dynamics, measured by real-time RT-PCR (RT-qPCR), all animals of the mock-vaccinated group developed a highly productive virus infection upon contact with seeder pigs. Compared to individually inoculated seeder pigs, the peak of virus excretion was delayed toward the end of the first week after contact. Clearance, however, was achieved rapidly during the second week of observation (Fig. 2B).

The two WIV-vaccinated groups showed a nasal virus excretion course that was similar to the mock group until 5 dpc, but then virus shedding decreased more rapidly compared to the mock group. In contrast, nasal excretion of viral RNA in the LAIV and VSV replicon-vaccinated groups was significantly reduced (*p* < 0.01) by up to 10 Cq values (representing 3 orders of magnitude) at dpc 2–4 compared to all other groups. The group which had received the bat-IAV chimeric vaccine revealed the lowest excretion peaks and cleared the virus rapidly from 4 dpc onwards (Fig. 2B).

### Efficient inhibition of virus spread to lower respiratory tract tissues by VSV replicon vaccines

**Fig. 3:**
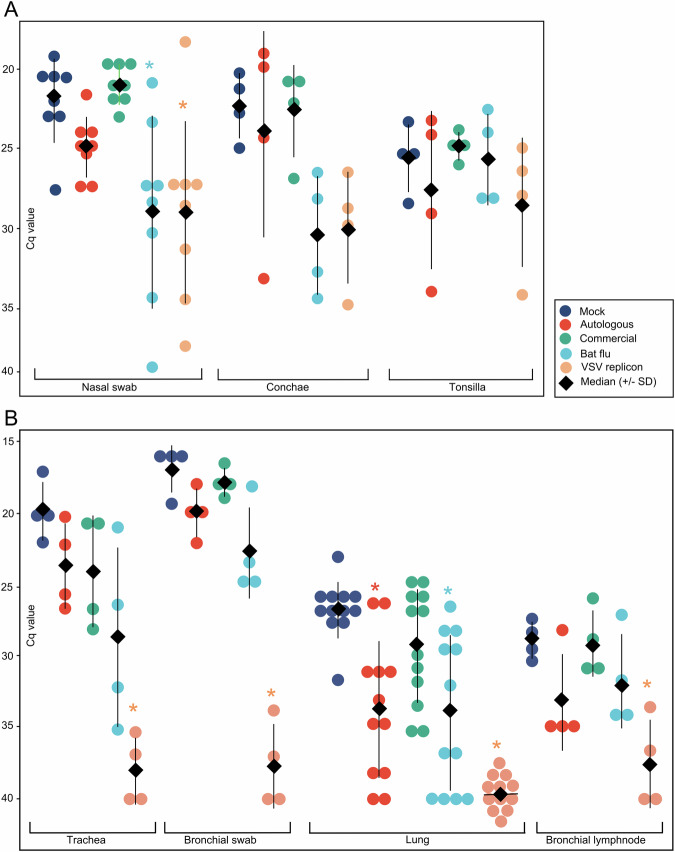
Nasal viral RNA excretion and spread of challenge virus RNA in tissues of the respiratory tract of four vaccinated piglets sacrificed at day 4 post contact challenge (dpc) with swIAV isolate H1pdmN2 (AI03362). Viral loads were measured by an M-specific RT-qPCR; Cq values are indicated on the Y-axis. **A** Upper respiratory tract; **B** Lower respiratory tract. Nasal swabs were obtained from all vaccinated group members at 4 dpc (*n* = 8); three separate lung locations were sampled per group member (i.e., a total of *n* = 12 per group). Error bars indicate the standard deviation of the median. Asterisks indicate highly significant differences (*p* < 0.01) of indicated group from Mock group (Supplementary Table 2; Kruskal–Wallis with Dunn–Bonferoni correction).

Four vaccinated and one seeder pig in each group were sacrificed at 4 dpc to examine the within-host spread of the challenge virus in tissues of the respiratory tract. On that day, all vaccines still showed nasal virus excretion (Figs. 2B, 3A). Analysis of the tissues of the upper respiratory tract tissue demonstrated lower nasal viral RNA loads in the conchae of both the bat-flu vaccine and VSV replicon groups (Fig. 3A). Viral RNA loads in tissues of the lower respiratory tract comprising trachea, the bronchial lumen (swab inserted into all large bronchial branches of the right lung lobe), lung tissues of three different lobar locations (*n* = 12 samples per group, left and right lung) and a bronchial lymph node mainly mirrored the results for the upper respiratory tract tissues (Fig. 3B). The highest mean viral RNA loads were observed in bronchial swabs, indicating the superior suitability of this sample for probing the tributary system of the lung as a whole. Animals of the mock group consistently showed the highest viral loads together with pigs that had received the inactivated and adjuvanted vaccines. Significantly lower virus loads were measured in the VSV replicon-vaccinated group which even remained completely negative in lung tissues (*p* < 0.001). Lower viral loads were also measured for the chimeric bat-flu vaccine group and for the autologous vaccine group, but only in lung and lymph node tissues. Values of the commercial multivalent WIV-vaccinated group remained indistinguishable from the mock-vaccinated one.

### Immunohistochemistry largely mirrored the reduced spread of the challenge virus in respiratory tract tissues of LAIV and replicon vaccine compared to WIV-vaccinated piglets

The extent of pulmonary atelectasis was documented for each lung lobe at 4 dpc (Supplementary Table 1). Macroscopically visible atelectases were found in 3 out of 5 seeder pigs and one bat-IAV vaccinated contact animal. The right middle lobe was consistently affected in these pigs. The changes rarely exceeded 5% per lobe, only one seeder showed 30% atelectasis.

For immunohistochemistry, the nose, trachea, and three different areas of the lung were tested for the presence of the IAV matrix-1 protein. Based on RT-qPCR results, two animals with the highest viral loads in the lungs (mock, commercial multivalent WIV, autologous WIV, bat-IAV, VSV replicon), trachea or nose were selected from each group. Viral antigen was detected in all groups at variable levels. The nose and trachea were positive in all vaccinated piglets except for the VSV replicon group. Compared to that, antigen detection in the lungs differed. While viral antigen was largely absent in the lungs in the VSV replicon and autologous group, it could be detected in varying amounts in bronchial respiratory epithelial cells in at least one lung sample in the other vaccine groups. In more detail, in the bat LAIV-vaccinated animals, antigen was detected less abundantly (oligofocal) compared to the commercially multivalent WIV-vaccinated pigs or the mock group (multifocal). Histopathological results are summarized in Supplementary Fig. 2.

### Antibody kinetics reflect constant decay of MDA during the vaccination phase and anamnestic responses following the challenge

**Fig. 4:**
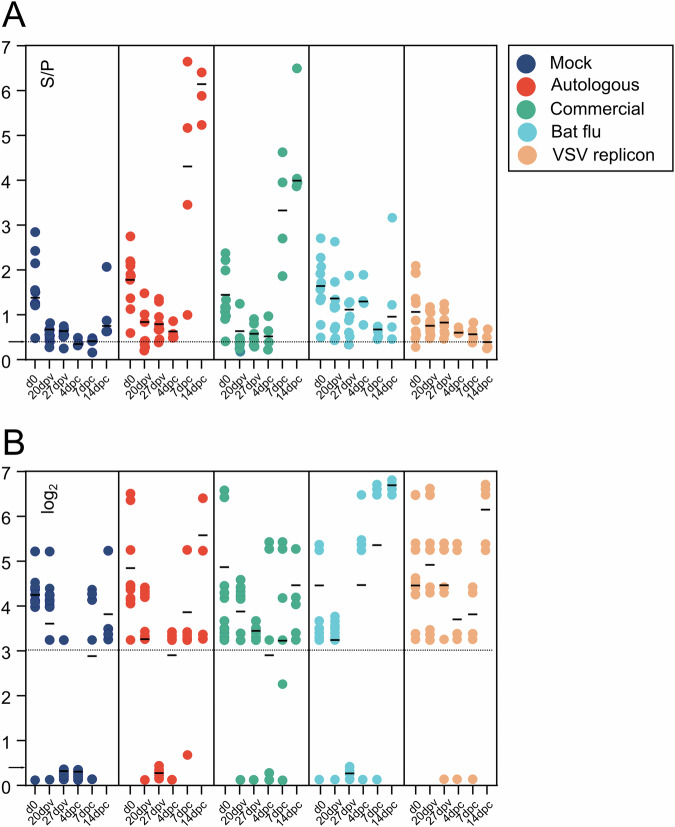
Influenza A virus-specific serologic immune responses. Measurements were achieved by **A** a nucleoprotein-specific indirect ELISA (ID-VET NP) and **B** by a hemagglutination inhibition assay using the swIAV H1pdmN2 isolate AI03362 as antigen. A - S/*P* value >0.4 is regarded as positive (dashed line), B – Titers of 3 log2 (1:20) are considered specifically positive. Error bars indicate the standard deviation of the median. d0 – Serum sample obtained at day 28 of age before vaccination at day 35; 20, 27 dpv - 3 weeks after first and 1 week after booster vaccination; 4, 7, 14 dpc – days post contact challenge (1 week after the booster vaccination). Dashed line – **A** threshold of S/P value = 0.4, **B** threshold of 1:20 (log_2_ 4,3).

Serum samples were examined by a generic blocking ELISA targeting the NP antigen and by hemagglutination inhibition (HI) assays using antigen of the H1pdmN2 isolate AI03362. As shown in Fig. 4a, all piglets had NP-specific antibodies at 28 days of age when the first sample was obtained (d0). These titers declined continuously during the vaccination phase which was most evident in the mock and VSV replicon groups (since these animals received vaccines lacking the swIAV NP antigen). In the other vaccine groups, a clear-cut serological NP response following vaccination until day 27 post vaccination (27 dpv) was not evident although in the bat-flu vaccine group NP antibodies remained at a slightly elevated level. Following contact exposure onwards from 63 days of age an exponential increase of NP-specific antibodies was evident in the groups that had been immunized with WIV (Fig. 4A). In other groups, single animals seroconverted as indicated by ELISA while the median value of NP antibodies decreased further in the VSV-immunized pigs, in line with the observation that challenge virus replication was efficiently inhibited by this vaccine.

A comparable picture emerged when examining the same sera by homologous HI assays (Fig. 4B). Median titers of HI antibodies decreased continuously until contact exposure. The exception are animals of the bat-IAV and replicon vaccine groups in which median titers stayed elevated. A steep increase in HI titers was evident by the end of the challenge phase (14 dpc); the exponential increase was most prominent in the bat-IAV and replicon groups but less pronounced in the two WIV groups.

## Discussion

Vaccination remains the main tool to combat swIAV infections in pigs. Here we compared the efficacy of two WIVs with a bat flu-based LAIV and a VSV replicon vaccine in an experimental setting with piglets from the same enzootically infected pig holding and a challenge virus that has been isolated from that holding. If commercial farms vaccinate against swIAV at all, it is usually only the sows, and WIV are being used. This vaccination concept at least accomplishes the protection of sows against swIAV-related disease. While early clinical protection of piglets is achieved by WIVs, due to interference with MDA infection and shedding are not prevented^1,17^. Sufficient herd immunity to suppress enzootic swIAV infections yet is unlikely to be generated when vaccinating just a small sector, i.e. the sows, of the herds’ population. All swIAV vaccines currently commercially available on the European market are WIV-based. Inactivated vaccines face problems in inducing broadly protective and sustained immunity as described for human seasonal influenza vaccines. Although adjuvants in swIAV vaccines for pigs, in contrast to human seasonal subunit vaccines, have a beneficial effect in broadening protection^16^, novel swIAV variants are constantly emerging in the field, and enzootically infected pig holdings accelerate this risk^22,41^. The ultimate goal of this study, therefore, was to test the vaccines´ efficacy in piglets despite the presence of specific antibodies.

As piglets constitute an important driver of sustained swIAV circulation in large holdings, we primarily aimed at this age class. Antibodies detected here in piglets most likely had a maternal origin as they rapidly declined over time, which is not expected of an actively acquired antibody response; this effect was most evident in the NP-specific ELISA in piglets receiving vaccines that did not contain NP (LAIV bat-flu and VSV replicon). Although MDAs provide clinical protection, even newborn piglets with high MDA activity are not reliably protected against infection and even represent an important amplifying host for swIAV in the herd^1,17,42^. Improved, and above all, actively acquired immunity in the age group of young piglets, therefore, appears desirable in order to interrupt continuous virus circulation in large herds. Yet, interference with maternally transmitted immunity poses a major obstacle when trying to induce active immunity against swIAV in very young piglets^43^. WIVs appear to be particularly prone to this interference, as shown previously^44,45^. Recent data suggested that single-dose WIV vaccination of young piglets may even foster sustained swIAV circulation in large holdings^46^. In addition, the role of antigenic mismatches between WIV vaccines and the circulating viruses remains to be determined: In the United States, but not in Europe, even vaccine-associated enhanced respiratory disease in weaned piglets with MDA has been observed upon heterologous challenge^47^.

Since serological options are insufficient to define protection, the challenge test remains the best possible alternative to test for the presence and resilience of vaccine-induced immunity in MDA-positive piglets. In general, it has been proven to be difficult to develop a challenge model for swIAV that can essentially be based on a clinical readout. Only highly artificial systems, such as the deep intratracheal application of a high-titer challenge virus suspension or the flooding of aerosol chambers with high-titer virus aerosols yielded clinically interpretable results for some virus strains^48,49^. Yet, the intranasal application of high-titer virus suspensions routinely used in previous studies still represents an artificial system that potentially skews the natural way of infection. To overcome this obstacle, infected seeder pigs have been successfully used to mimic natural infection routes in challenge experiments^45,50,51^, and seeder pigs have been employed for the current study. Although this approach is associated with an increased risk of uneven and quantitatively undefined virus exposure^52^, nasal virus excretion dynamics of our challenge control group clearly indicated that all animals exposed to seeder pigs became infected simultaneously and developed a productive infection with seroconversion rates that were indistinguishable from those of intranasally inoculated seeders (Figs. 2, 3). Similar patterns were observed in the vaccinated animal groups, in particular those which were not adequately protected. As parallel, longitudinal studies in this specific pig herd showed a cluster of natural swIAV infection in piglets aged 4–8 weeks^53^, we attempted to recreate this situation in the herd as closely as possible and exposed the animals to a challenge at 7-8 weeks of age even at the risk that the short 7 days interval since the booster vaccination could have resulted in a still suboptimal immune response. An alternative to the short interval until the challenge would have been an earlier start of the vaccination scheme. However, piglets were not weaned before 28 days of age and application in the field (i.e., the holding of origin) of vaccines containing unlicensed replication-competent genetically modified organisms (i.e., the chimeric bat virus and the VSV replicon) is prohibited by the German genetic engineering law.

Although piglets were used that had been raised until weaning in a commercial swine herd, i.e., did not have SPF status, hardly any clinically measurable changes were induced by the H1pdmN2 challenge. This also applies to the macroscopic findings, based on the fact that even not all of the seeder animals and the mock-vaccinated control group showed lesions in the lungs. Thus, protective effects had to be assessed by measuring viral RNA loads in nasal swabs and in organs of the respiratory tract.

Virus loads measured here show that none of the vaccines used were capable of inducing sterile immunity in the individual animal; in contrast, each vaccine remained susceptible to some degree to infection and (limited) virus replication following seeder pig exposure. This could, in part, be due to the early challenge date just one week following the second vaccination. At that time, serological responses of the vaccinees did not show a clear booster effect (Fig. 4). However, this may be in part also related to the presence of MDA and their interference at least during primary vaccination. Compared to WIVs, LAIV can offer enhanced protection as it imitates natural infection, is administered intranasally, infects the upper respiratory tract, and stimulates both humoral and cell-mediated immunity^54^. As an advantage of the intranasal application of LAIV the induction of mucosa-associated specific immunity is mentioned in first place^55–58^. Their effectors are expected to act in the sense of an immune exclusion by immediately neutralizing or at least limiting the replication and spread of incoming pathogens. In this study, we did not investigate the presence of mucosa-based H1N2-specific antibodies (i.e., IgA) or of tissue-resident T cell populations as the amount of putative mucosal immunity would not have had an influence on the dose of challenge virus to which pigs were exposed. Instead, we put the focus on the aspect of comparing the overall efficacy of different vaccines in preventing virus excretion. In terms of reduction of nasal virus excretion the effect of the bat-IAV vaccine, which was the only intranasally applied vaccine in our study, was striking and unsurpassed by any other vaccine (Fig. 2B). This likely indicates effects of mucosal immunity as shown for other bat-IAV chimeras^30^. Nevertheless, virus replication was not fully abrogated even after homologous challenge and, therefore, increased excretion levels must be expected following heterologous challenge. It remains to be determined if the chimeric bat-IAV is replicating sufficiently or whether it is over-attenuated^29^.

The intramuscular immunization of piglets with the VSV replicon vaccine also led to significantly reduced replication of swIAV in the upper respiratory tract (Fig. 3A), with consequent reduced nasal excretion of swIAV (Fig. 2B). However, the most impressive effect by vaccination with the replicon vaccine was the drastically reduced virus replication in the lower respiratory tract to almost undetectable levels (Fig. 3B), even though a single cell of one lung sample was found to be antigen positive in immunohistochemistry (Supplementary Table 2B). Numbers of animals and samples are limited and tissue locations are not fully congruent between IHC and RT-qPCR samples. This may account for observed differences in lung tissue samples of the autologous and the replicon groups. As both techniques represent different perspectives, it still can be concluded that the viral load found either via RT-qPCR or IHC in the lungs is close to the detection limits of either method.

The VSV replicon vaccine was the only vaccine tested that induced significant levels of hemagglutination inhibiting (HI) serum antibodies at 27 days post vaccination (Fig. 4B). As serum IgG can be secreted into the respiratory tract, in particular into the lung tissue^59^, these antibodies may explain in part the protective features of this vaccine. Furthermore, the VSVΔG(N2) component of the vaccine may have also contributed to protection. We did not investigate the level of sialidase-inhibitory antibodies induced here, however, our previous studies have shown that VSV replicon-driven expression of NA antigen alone can protect animals from IAV infection^38,60^. Finally, VSV replicons were previously shown to induce specific T cell immune responses in vaccinated pigs, which may also contribute to protection and virus clearance^36^. It would be interesting to assess the use of combined simultaneous or consecutive use of the VSV replicon and the LAIV bat-IAV evaluated here. Such concepts could even be instrumental when attempting to broaden cross-protective immunity by heterologous prime-boost strategies as recently suggested for WIVs^61–63^.

Regarding the putative impact of the antigenic match between vaccine and field virus strains, here only the two WIV vaccines can be compared which differed in this parameter. The autologous WIV based on the homologous antigen presented with advantages over the commercial heterologous WIV as far as reduction of challenge virus in deeper tissues of the respiratory tract was concerned (Fig. 3B). Compared to the Mock group, both WIVs shortened the time period of nasal virus excretion with similar efficacy which, nevertheless, was inferior to the effects of the replication vaccines (Fig. 2B).

In conclusion, the present vaccination trial hints at a superior efficacy of bat-flu LAIV and VSV swIAV vaccines in reducing nasal virus excretion and, in particular for the replicon vaccine, limiting spread of challenge virus to lower respiratory tissues in young piglets with MDAs.

These findings highlight the potential of LAIV and multiplication-incompetent replicon vaccines as a valuable tool for controlling and preventing swIAV outbreaks in pig populations, ultimately contributing to the overall health and productivity of the swine industry as well as to the “One Health” approach. Nevertheless, LAIV and RNA replicon vaccines might also require regular updates to closely match the antigens of circulating viruses. In this respect, replicon technology may prove more flexible and is affected with fewer biosafety concerns compared to bat-IAV chimeras. However, there is currently no evidence that the replacement of HA/NA in bat-associated influenza A viruses of subtype H17N10 could significantly increase the risk potential of these viruses (assuming that only HAs expressing a trypsin-sensitive cleavage site are used). Previous studies demonstrated that natural bat-IAV infections have not yet been associated with pathogenicity for humans, non-bat mammals, or other species. The distinctive advantage of bat chimeric influenza viruses is that reassortment with influenza viruses of birds and non-bat mammals is blocked, thereby preventing reversion to virulence. Another advantage of the VSV replicon vaccines is their efficacy in the presence of IAV-specific MDA, as infection by the replicon particles is mediated by the VSV G protein and not by the IAV envelope glycoproteins. A disadvantage of the replicon particles used here is that they have to be applied by injection, while a nasal/oral form of application would be ideal from an immunological perspective. From a practical point of view, individual nasal vaccine applications in the field are feasible in week-old piglets, but become challenging already in 3-week-old ones and virtually impracticable in elder pigs. Therefore, further studies are warranted also to (i) assess the long-term efficacy and safety of these vaccines in the field, (ii) compare new vaccination schemes including cross-boostering to broaden protection to heterologous virus strains, and (iii) consider adapted vaccination strategies in continuously infected holdings: Instead of vaccinating sows only, at least for some period of time, efficient vaccination of all piglets born using replication-competent vaccines might be essential to build up resilient and high-level population immunity to interrupt continuous swIAV transmission chains.

## Methods

### Swine influenza A virus

The virus strain A/swine/Germany/2022AI03362/2022 (abbr. AI03362) of subtype H1pdmN2 has been isolated from a nasal swab of a piglet at an enzootically infected German swine herd in western Germany using a swine testicle (ST) cell line as previously described^64^. Following two further passages in ST cell cultures, the stock virus was generated, titrated, and kept until −80 °C until further use. The stock virus was fully sequenced (accession number EPI_ISL_17646249) and HA genotyping as clade 1A.3.3.2/pdm (II-like) confirmed. Virus titration was performed in MDCK II cells. Permission for repeated sampling in the holding was granted by an independent ethical committee (project 33.19-42502-04-22-00225).

### Vaccines

Based on the stock virus material itself or on the genome sequences obtained from it, replication-competent experimental vaccines were produced:

#### VSV replicons

Recombinant VSV replicons expressing the haemagglutinin (HA) or neuraminidase (NA) of AI03362 were generated according to a previously published procedure^40^. Recombinant VSV replicons were approved by the Swiss Genetic Engineering Authority under license number A202815-00 and reported to the State Office for Health and Social Affairs of Mecklenburg-Western-Pomerania, Germany. The recombinant VSVΔG-H1 and VSVΔG-N2 vectors were propagated on a complementing helper cell line (BHK-G43)^65^. The cell culture supernatant was collected at 24 h pi and cleared by low-speed centrifugation. The virus replicon particles were pelleted by ultracentrifugation (105,000×*g*, 60 min, 4 °C) and resuspended in phosphate-buffered saline, pH 7.4 (PBS), and stored in aliquots at −80 °C. For determination of the infectious virus titer, an aliquot was thawed and titrated on BHK-21 cells as described^40^.

#### Bat influenza chimeric virus

The chimeric bat influenza virus was approved by the Regional Council of Baden-Württemberg, Germany (license number SSI2-UNI.FRK.05.23/05.18/05.22.) and the State Office for Health and Social Affairs of Mecklenburg-Western-Pomerania, Germany (LAGuS3021_4/11.5.17), and was generated as described previously^33,66,67^. Briefly, the HA coding region of swIAV AI03362 was flanked at the 3′- and 5′-terminal ends by the putative *cis*-acting terminal packaging sequences from A/little yellow-shouldered bat/Guatemala/164/2009 (H17N10) (nucleotides 1 to 131 for the 3′ noncoding region and nucleotides 1621 to 1784 for the 5′ noncoding region). Similarly, the NA coding region of swIAV AI03362 was flanked by the putative *cis*-acting terminal packaging sequences from H17N10 at the 3′ and 5′ ends (nucleotides 1 to 122 for the 3′ noncoding region and nucleotides 1254 to 1390 for the 5′ noncoding region). To prevent premature protein synthesis, all ATG start codons located within the putative terminal packaging sequences were mutated to AAG. The six internal gene segments (PB2, PB1, PA, NP, M, and NS) were from A/little yellow-shouldered bat/Guatemala/153/2009 (H17N10) (GenBank accession numbers CY103873, CY103874, CY103877, CY103875 [PA_S550R_], CY103880, and CY103879 [M1_D156N_ and M2_N31S;T70A_]). The recombinant bat influenza chimera was plaque-purified on MDCK II cells and then used for stock generation. At 48 h post infection virus supernatants were collected and stock titers were determined via a plaque assay on MDCK II cells. Two bat-IAV chimera stocks were generated reaching titers of 3.8 × 10^7^ PFU/mL (stock 1) and 8×10^6^ PFU/mL (stock 2). Importantly, these stock titers were comparable to that of the parental swIAV on MDCK II cells (data not shown).

#### Monovalent autologous inactivated and adjuvanted whole virus vaccine

The autologous vaccine was produced as an inactivated whole virus vaccine, containing the homologous H1pdmN2 isolate AI03362. The autologous vaccine was designed for a dosage of 1 ml/animal. The virus concentration before inactivation is >256 HAU/ml. A polymer was used as adjuvant (Ictyolane 50®, IctyoDev, France). A polymer-adjuvanted mock vaccine based on the supernatant of uninfected ST cell cultures was produced under the same conditions.

#### Multivalent heterologous inactivated and adjuvanted whole virus vaccine

The commercial licensed vaccines used were the trivalent Respiporc FLU3 containing antigens of the subtypes H1huN2 (A/swine/Bakum/1832/2000), H3N2 (A/sw/Bakum/IDT1769/2003) and H1avN1 (A/swine/Haseluenne/IDT2617/2003) as well as the monovalent Respiporc FLUpan H1N1 containing the subtype H1pdmN1 (A/swine/A/Jena/VI5258/2009). These were provided by the producer, Ceva Santé Animale, Libourne, France.

### Animal experiments

#### Ethics statement

The animal experiments were approved by an ethics committee at the State Office for Agriculture, Food Safety and Fishery of the Federal State of Mecklenburg-Western Pomerania, Germany by license LALFF M-V 7221.3-1-001/23. Animal numbers followed the specifications of the European Pharmacopoeia (Ph. Eur., Monograph on Porcine Influenza Vaccine (inactivated; 01/2017:0963) where possible.

#### Animals (origin)

A total of 50 German landrace weaner pigs at 28 days of age were obtained from the enzootically infected pig holding in Lower Saxony, Germany, that yielded virus isolate AI03362. All purchased pigs tested negative in nasal swabs for swIAV RNA by RT-qPCR at 28 days of age at the holding and after arrival at the FLI at 35 days of age (prior to vaccination). Longitudinal studies in this pig holding gave evidence that the earliest time point of swIAV infections in piglets was at 4 weeks of age (Supplementary Table 3)^53^. Thus, productive swIAV infections should have been detectable but were excluded here. IAV nucleocapsid protein-specific antibodies in serum by ELISA (ID Screen® Influenza A Nucleoprotein Swine Indirect, IDVET, Germany) gave positive results for all piglets. As infections with both Porcine Circovirus-2 (PCV2) and Porcine Respiratory and Reproductive Syndrome Virus (PRRSV) potentially exert immunosuppressive effects and might, therefore, influence the outcome of swIAV vaccination and challenge, nasal swabs and serum samples were investigated in RT-qPCRs to exclude the presence of both pathogens^68,69^. In none of the animals either PCV2 or PRRSV has been detected in samples at 28 days of age. After that date, the animals were reared at FLI’s BSL2 or BSL3 (bat-IAV chimeric vaccine group only) stable environments.

#### Experimental vaccination and challenge

Five groups of eight piglets each were assembled randomly and assigned to separate stable units. Eight additional pigs received no treatments and were kept separate from group 6. The animals were vaccinated according to Table 1. At day 35 of life, groups 2–5 received a different vaccine and group 1 was sham-vaccinated (control group). Group 4 received the bat-flu chimeric virus at a dose of 10^6^ TCID_50_/ml intranasally using a mucosal atomization device (Wolfe Tory Medical, USA); this group was the only one kept at BSL3 stable facilities. Groups 1–3 and 5 were vaccinated by intramuscular injection of the indicated dose/volume into the upper neck region (Table 1). Animals of group 5 were immunized by separate injection of the VSVΔ-H1 and VSVΔ-N2 replicon particles into the neck muscle (2 ml of each replicon, 10^8^ ffu/ml). The VSV replicon vaccine was used without adjuvant. Group 1 was used as control and received a vaccine similar to the autologous WIV but lacking the viral antigens. At day 56 of age, a booster application of the same vaccines was given to groups 1–5. Seven days later, i.e., at day 63 of age, pigs were exposed to the challenge virus AI03362 (H1pdmN2) isolated from the holding of origin of the piglets. In order to mimic natural infection, a contact challenge model was used. To this end, ten “seeder piglets” of the same age and origin (group 6) were individually inoculated intranasally at 61 days of age with 10^6^ TCID_50_ of isolate AI03362 in 1 ml cell culture supernatant using a mucosal atomization device. After 48 h, each group of vaccinated piglets were exposed to two seeder pigs to initiate natural infection by contacts or aerosol. This day was counted as day 1 of contact (doc). At 4 days post contact challenge (dpc), four inoculated pigs and one seeder pig per group were humanely euthanized, followed by exsanguination, followed by dissection of the respiratory tract. At 14 dpc, all remaining animals were autopsied. The study design is summarized in Fig. 1.

**Table 1:** Vaccinated groups (1–5) of piglets derived from an enzootically infected herd and challenged by exposure to seeder pigs infected with swIAV isolate H1pdmN2 (AI03362)

Study group	Group size (*n*)	Vaccine	Application mode	Vaccine dose	Challenge dose	Exposure to challenge virus
				and volume	and volume	
1	8	Mock (control)	i.m.	1 ml	-	Contact to seeder
2	8	Autologous WIV	i.m.	1 ml per vaccine	-	Contact to seeder
				(2 ml in total)	-	
3	8	Multivalent heterologous WIV	i.m.	1 ml per WIV	-	Contact to seeder
				(2 ml in total)	-	
4	8	Bat-IAV chimeric LAIV	i.n.	2 ml (10^6^ TCID_50_)	-	Contact to seeder
5	8	VSV replicon	i.m.	1 ml per vaccine (2 ml in total)	-	Contact to seeder

*LAIV* live-attenuated influenza vaccine, *WIV* whole virus inactivated adjuvanted vaccine, *i.m*. intramuscular, *i.n*. intranasal, *n.a*. not applicable.

#### Clinical observation and postmortem sampling

Clinical scores (according to parameters detailed in the Supplementary Information file) and body temperature measurements were recorded daily throughout the observation period. During post mortems, a bronchial swab of all large bronchi of the right lung and tissue samples of nasal conchae, trachea, and three lung areas (cranial and caudal part of left cranial lobe, left medial lobe), and a tracheobronchial lymph node were collected to determine viral load by RT-qPCR.

### Virological testing

Nasal swabs were taken at the indicated time points (Fig. 1) and placed into 2 ml tubes containing 1 ml of Eagle’s Minimal Essential cell culture medium (EMEM) supplemented with 100 U/ml penicillin and 100 µg/ml streptomycin (sample medium). Swab samples were vortexed and centrifuged to remove debris. Tissue samples were collected from all pigs at the postmortem stage, placed in 2 ml tubes with 1 ml sample medium, and homogenized by using a 5 mm thick steel ball and a TissueLyser. The material was then centrifuged, and the supernatants were aliquoted. Automated RNA/DNA extraction was achieved with the King Fisher Flex Purification System (Thermo Fisher Scientific) and the NucleoMag® Vet Kit (Macherey-Nagel GmbH & Co. KG, Dueren, Germany) according to the manufacturer’s instruction. For initial IAV testing, all samples were analyzed in a modified generic Matrix RT-qPCR^70^. Samples yielding a cq value of ≤39.9 were considered IAV-positive. In addition to IAV testing, the presence of porcine reproductive and respiratory syndrome (PRRSV) as well as of porcine circovirus type 2 (PCV2) in nasal swabs and serum samples from day 0 prior to the vaccination were investigated in RT-qPCR assays. For PRRSV, a modified method based on a publication by Kleiboeker, Schommer, Lee, Watkins, Chittick, and Polson^69^ consisting of PCR systems for both the North American and the European genotypes was used. The detection of PCV2 was based on a publication by Brunborg, Moldal, and Jonassen^68^. All RT-qPCR’s reactions were prepared with the AgPath-ID^TM^ One-Step RT-PCR kit (Thermo Fisher Scientific, United States) by using a Biorad CFX96 Real-Time Cycler (Biorad, Germany). For the detection of PCV2, a DNA virus, the RT step was omitted.

### Serological testing

Blood samples were collected at the indicated time points (Fig. 1) accessing the jugular vein. Blood was allowed to clot before centrifugation at 3000 rpm for 10 min, the serum then recovered, heat-inactivated for 30 min at 56 °C and afterwards frozen at −20 °C until further use. Sera were first screened using an indirect enzyme-linked immunosorbent assay (ELISA) detecting generic antibodies against the nucleoprotein (NP) (ID Screen® Influenza A Nucleoprotein (NP), swine indirect ELISA; ID, Grabel, France). The cut-off values for sample to negative (S/N) ratios were interpreted as recommended by the manufacturer: S/N ≤ 0.4 negative; S/N ≥ 0.4 positive. In addition, all samples, after neuraminidase treatment, were further examined by hemagglutinin inhibition (HI) using the AI03362 H1pdmN2 vaccination and challenge antigen as described elsewhere (WOAH, 2023).

### Histopathology and immunohistochemistry

A full autopsy was performed on all animals under BSL2 or BSL3 conditions. The extent of pulmonary atelectasis was documented for each lung lobe (percentage of affected area). Tissue samples of nasal conchae, trachea, and three lung areas (cranial and caudal part of left cranial lobe, left middle lobe) were collected at 4 dpc and fixed in 10% neutral-buffered formalin. After trimming and paraffin embedding, hematoxylin and eosin (HE) staining was applied to 2–3-μm-thick slices. As stated previously^71^, consecutive slides were treated for immunohistochemistry using the standardized avidin-biotin-peroxidase complex method. The primary monoclonal antibody HB-64 targeting the M1 protein of IAV, diluted 1:200, was applied overnight at 4 °C (M21C64R3, ATCC, Manassas, VA). The secondary antibody (goat anti-mouse, biotinylated; BA-9200-1.5, Vector Laboratories, Burlingame, CA, USA), diluted 1:200, was applied for 30 min at room temperature. All sides were scanned using a Hamamatsu S60 scanner, evaluation and description was done using the NDPview.2 plus software (Version 2.8.24, Hamamatsu Photonics, K.K. Japan) by a board-certified pathologist in a blinded fashion. From each vaccine group, animals (*n* = 2) with the highest viral genome loads in the lungs (mock, commercial multivalent WIV, autologous WIV, bat-IAV) or nasal conchae (VSV replicon) were selected for immunohistochemistry.

### Statistical analyses/Software

Graphics and statistics were produced using R (RStudio version 2023.09.1, packages ggplot2, ggline, ggpar, etc.) and GraphPad prism version 9.0.0 for Windows (GraphPad Software, La Jolla, CA). Figure 1 was created with BioRender.com and licensed by the company under agreement number CL26YA977R. Significance levels were calculated by the Kruskal–Wallis test with Dunn–Bonferroni corrections (Supplementary Table 2).

### Supplementary information

Supplementary Information

## Data Availability

The datasets used and/or analysed during the current study, if not already presented in the main text or in the Supplementary Information file, are available from the corresponding author on reasonable request.
