# Supplementary material for: Reassortment incompetent live attenuated and replicon influenza vaccines provide improved protection against influenza in piglets
Source: NPJ Vaccines. 2024 Jul 13;9:127. doi: 10.1038/s41541-024-00916-x (PMC11246437; doi:10.1038/s41541-024-00916-x)
Supplement: Supplementary file 1 — Supplementary Information [file 41541_2024_916_MOESM1_ESM.pdf]

Supplementary Figure 1. Median rectal temperature of pigs challenged with swIAV isolate H1pdmN2 AI03362 by contact to inoculated seeder animals. Dashed line shows the 40°C fever threshold.

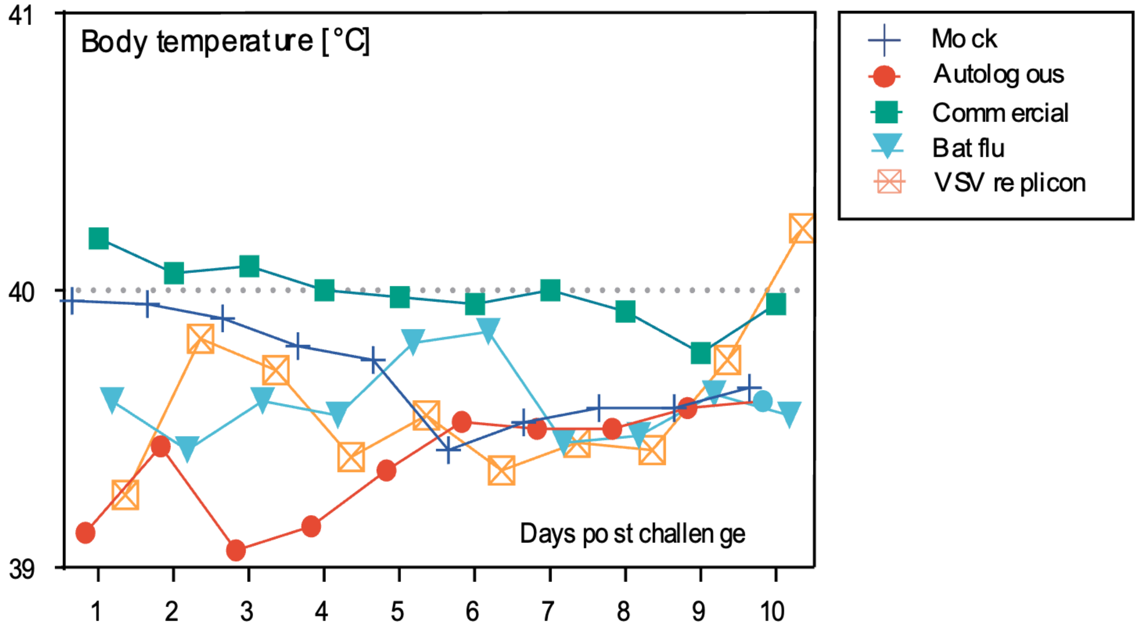

Supplementary Figure 2. Histopathological results of representative WIV and LAIV vaccinated pigs after challenge infection with H1pdmN2. A) Immunohistochemical results of the nose, trachea and lung of individual pigs selected based on positive viral genome detection. (+) = positive, (-) = negative for viral matrix protein. B) Immunohistochemistry of the lung illustrating IAV matrix-1 protein-positive bronchial respiratory epithelial cells (arrow) and foci of alveolar cells (VSV-LAIV only, inlay) in the different groups. Scale bar = 50 µm.

A

Study group	Nose	Trachea	Lung 1	Lung 2	Lung 3
Mock (control)	+	+	-	+	-
Mock (control)	+	+	+	-	-
Autologous WIV	+	+	-	-	-
Autologous WIV	+	-	-	-	-
Multivalent heterologous WIV	+	+	+	+	+
Multivalent heterologous WIV	+	+	-	+	+
Bat-IAV chimeric LAIV	+	+	+	+	-
Bat-IAV chimeric LAIV	-	+	+	-	+
VSV replicon	-	-	-	+	-
VSV replicon	-	-	-	-	-

B

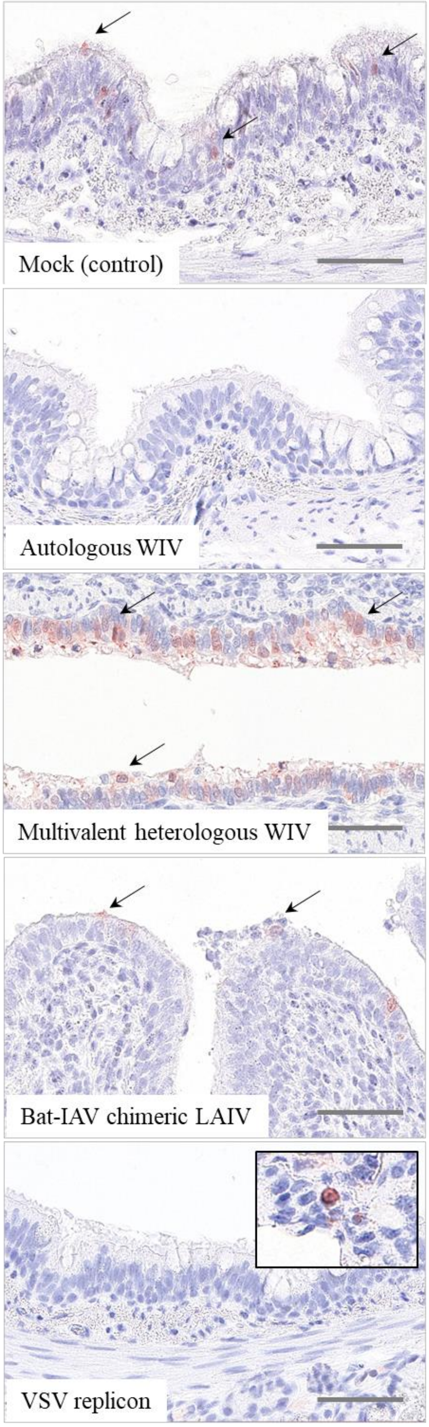

Supplementary Table 1. Post mortem findings, 4 dpc, pulmonary atelectasis given in percent afftected area (%).

**Study group**	**Status**	**pulmonary atelectasis (%) and affected lung lobe**
**Mock (control)**	Seeder	<5 % cranial part of left cranial lobe, right middle lobe, right caudal lobe
	vaccinated contact	-
	vaccinated contact	-
	vaccinated contact	-
	vaccinated contact	-
**Autologuous WIV**	Seeder	-
	vaccinated contact	-
	vaccinated contact	-
	vaccinated contact	-
	vaccinated contact	-
**Multivalent heterologous WIV**	Seeder	-
	vaccinated contact	-
	vaccinated contact	-
	vaccinated contact	-
	vaccinated contact	-
**Bat-IAV chimeric LAIV**	Seeder	<5 % caudal part of left cranial lobe, right middle lobe
	vaccinated contact	-
	vaccinated contact	-
	vaccinated contact	-
	vaccinated contact	<5 % right middle lobe
**VSV replicon**	Seeder	30% right middle lobe
	vaccinated contact	-
	vaccinated contact	-
	vaccinated contact	-
	vaccinated contact	-

Supplementary Table 2. Significance levels calculated by the Kruskal-Wallis test using the Dunn-Bonferoni correction for pairwise comparisons. Highly significant differences of vaccine groups versus the Mock group are indicated by asterisks in Figures 2 and 3 in the main text.

	Lower respiratory tract (Fig. 3b)				Upper respiratory tract		Nasal swabs by day post challenge (Fig. 2b)					
**Vaccine groups**	**Trachea**	**Bronchial swab**	**Lung**	**Lymphnode**	**Conchae**	**Tonsil**	**2**	**3**	**4**	**5**	**6**	**7**
Bat flu-Mock	0.0364	0.03138	0.002964	0.3095	0.0486	0.6113	0.1262	0.003633	0.001152	0.001886	0.002807	0.00207
Bat flu-WIV Comm	0.5108	0.1201	0.03092	0.4026	0.0486	0.3095	0.0004001	0.01311	0.02762	0.01018	0.01207	0.3858
Bat flu-VSV replicon	0.135	0.1884	0.01698	0.08297	0.8577	0.7423	0.9318	0.8979	0.5637	0.2562	0.232	0.05193
Bat flu-WIV auto	0.4026	0.5499	0.7723	0.4731	0.05583	0.5108	0.01708	0.0795	0.000728	0.03144	0.1693	0.08285
Mock-WIV Comm	0.1513	0.5499	0.3894	0.8577	1	0.6113	0.04436	0.6689	0.2947	0.5907	0.6326	0.02691
Mock-VSV replicon	0.0003345	0.0005254	1,12E-04	0.005958	0.03647	0.8577	0.1488	0.005435	0.007515	0.0486	0.073	0.2558
Mock-WIV auto	0.2093	0.1201	0.007177	0.08297	0.9049	0.2437	0.3922	0.2482	0.8979	0.339	0.1066	0.1784
WIV Comm-VSV replicon	0.03138	0.00411	0.000005484	0.01015	0.0486	0.4918	0.0005513	0.01866	0.1041	0.1515	0.1886	0.2817
WIV Comm-WIV auto	0.8577	0.3388	0.06165	0.1201	0.9049	0.09414	0.2481	0.4672	0.2395	0.6757	0.2562	0.3858
VSV replicon-WIV auto	0.01972	0.05574	0.007438	0.3095	0.03647	0.3239	0.02148	0.1041	0.005088	0.3097	0.8577	0.8342

Significant (p=0,049-0.01)

highly signifcant (p<0,01)

Supplementary Table 3. Detection of swIAV in nasal swabs of piglets in the selected pig herd by RT-qPCR at different weeks of age. A herd with at least one positive sample (cq ≤30) is marked in bold.

	age (week(s))	***herd tested positive (cq ≤30)***
suckling piglets	1	-
	2	-
	3	-
weaned piglets	4	**yes**
	5	**yes**
	6	**yes**
	7	-
	8	**yes**
	9	-
	10	-
